# Peer Review of “COVID-19 and Cybersecurity: Finally, an Opportunity to Disrupt?”

**DOI:** 10.2196/29096

**Published:** 2021-05-06

**Keywords:** COVID-19, cybersecurity, challenges and disruption, data protection, privacy, health data

*This is a peer-review report submitted for the paper "COVID-19 and Cybersecurity: Finally, an Opportunity to Disrupt?"*

## Round 1 Review:

Dear Authors,

Thank you for the opportunity to read and review this paper [1]. I found it very interesting, useful, and well-written. I have made some comments on how the paper, in my view, can be further enhanced. Please consider them as suggestions:

### Introduction

For reader-friendliness: provide a short explanation of abbreviations (eg, COVID-19) the first time they appear in the text.

I miss some references in a few sentences, such as “Within the literature, recommendations are that data analyses are initiated and concluded openly, in accordance with the law and with respect for privacy.”

I also think that this section can be expanded.

At the end, when you outline the focus of the study and possible implications, it would be interesting to learn more about in the way in which documentation can serve as a learning tool. I recommend adding examples of practical and theoretical implications.

### COVID-19, Cybersecurity, Privacy, Security, and Safety

I would like to see information on how we are dealing with misinformation and false news and groups resisting the response to the pandemic under the topic of Education/Media, as this is an important issue the European Union and other countries are facing. Is this an EU or country-by-country initiative?

### Conclusion

The introduction to the conclusion is very similar to the beginning of the paper. The conclusion, to me, has the feel of being more of a summary than an actual conclusion. I would recommend narrowing down this section.

### Language

The language is overall very good. Some editing is recommended to ensure consistency in terminology and structure.

### Limitations

The paper is very positive without discussing limitations, challenges, and problems sufficiently. I suggest either incorporating this discussion throughout or providing a section at the end that also discusses the effectiveness of the measures as understood to date as well as current and future anticipated challenges.

### References

References should be expanded to include current literature. You should also add more references. Please include the following references: [2-6].
